# Antennal and palpal sensilla of three predatory *Lispe* species (Diptera: Muscidae): an ultrastructural investigation

**DOI:** 10.1038/s41598-021-97677-7

**Published:** 2021-09-15

**Authors:** Genting Liu, Qike Wang, Xianhui Liu, Xinyu Li, Xiunan Pang, Dong Zhang

**Affiliations:** 1 School of Ecology and Nature Conservation, Beijing Forestry University, Qinghua East Road No. 35, Mailbox 162, Beijing, 100083 China; 2 School of BioSciences, The University of Melbourne, Victoria, 3010 Australia; 3 University of California Davis, Davis, CA 95616 USA

**Keywords:** Taxonomy, Sexual selection

## Abstract

Antennae and maxillary palps are the most important chemical reception organs of flies. So far, the morphology of antennae and maxillary palps of flies of most feeding habits have been well described, except for that of relatively rare aquatic predatory species. This study describes sensilla on antennae and maxillary palps of three aquatic predatory *Lispe* species: *Lispe longicollis*, *L*. *orientalis* and *L*. *pygmaea*. Types, distribution, and density of sensilla are characterised via light and scanning electron microscopy. One type of mechanoreceptors is found on antennal scape. Mechanoreceptors (two subtypes) and one single pedicellar button (in *L*. *pygmaea*) are located on antennal pedicel. Four types of sensilla are discovered on antennal postpedicel: trichoid sensilla, basiconic sensilla (three subtypes), coeloconic sensilla and clavate sensilla. A unique character of these *Lispe* species is that the coeloconic sensilla are distributed sparsely on antennal postpedicel. Mechanoreceptors and basiconic sensilla are observed on the surface of maxillary palps in all three species. We demonstrated clear sexual dimorphism of the maxillary palps in some of the *Lispe* species, unlike most other Muscidae species, are larger in males than females. This, along with their courtship dance behaviour, suggest their function as both chemical signal receiver and visual signal conveyer, which is among the few records of a chemical reception organ act as a signal conveyer in insects.

## Introduction

Antennae and maxillary palps are the main chemical reception organs of flies on which numerous sensilla of various types can be found^1,2^. These organs play indispensable roles in the lives of flies in searching for food sources, mates, oviposition sites as well as other key life history stages^2–11^. Flies are under high selection pressure for receiving sufficient chemical signals and/or cues that are associated with their life history, such as searching for mates^12^, foods^13^ or hosts^14^, and this could influence the morphology of the antennae^15^. Flies have a wide range of feeding habits including saprophagy, phytophagy, parasitism, hematophagy and predatory^16,17^, making them ideal models for studying the adaptation of insect olfactory organs according to different olfactory requirements. It is well documented that flies of different feeding habits have different antennal shape and sensillar types^7–11^. Structure of antennae and maxillary palps, especially the distribution and morphology of sensilla have been documented in detail in saprophagy, phytophagy and parasitismflies^7,8,18,19^, but few researches have focused on the predatory flies.

The species of genus *Lispe* Latreille (Diptera: Muscidae) are among the relatively rare predatory flies, closely associated to aquatic and subaquatic habitats^20^. Adult *Lispe* flies are commonly found around the margin of ponds, lakes, streams or seashore and prey on various insects including several mosquito species, such as anopheline and chironomid^21,22^. Visual perception is comparatively more important for these flies in hunting for their flying preys than chemical cues, yet they should still rely on their antennae and maxillary palps for olfactory cues and signals. Therefore, it is expected that the olfactory perception requirements of *Lispe* flies are largely different from that of saprophytic and parasitic flies, and this presumably results in specific antennal morphology adaptations. For example, *Lispe neimongola* Tian *et* Ma^9^ has two conspicuous distinctions: the absence of coeloconic sensilla (Co) and enlarged spoon-like maxillary palps. It is unclear whether these morphological characteristics are common among other *Lispe* flies.

In this study, we describe the morphology of antennae, maxillary palps and sensilla located on them among three common *Lispe* species: *Lispe longicollis* Meigen, *L*. *orientalis* Wiedemann, and *L*. *pygmaea* Fallén^21,23,24^. Combined with the data of *L*. *neimongola*^9^, we compare the morphology of antennal and maxillary palps of *Lispe* with other Muscoidea species, in order to reveal their morphological characteristics adapted to the aquatic predatory life style.

## Results

### General description of the antenna and maxillary palp

**Figure 1:**
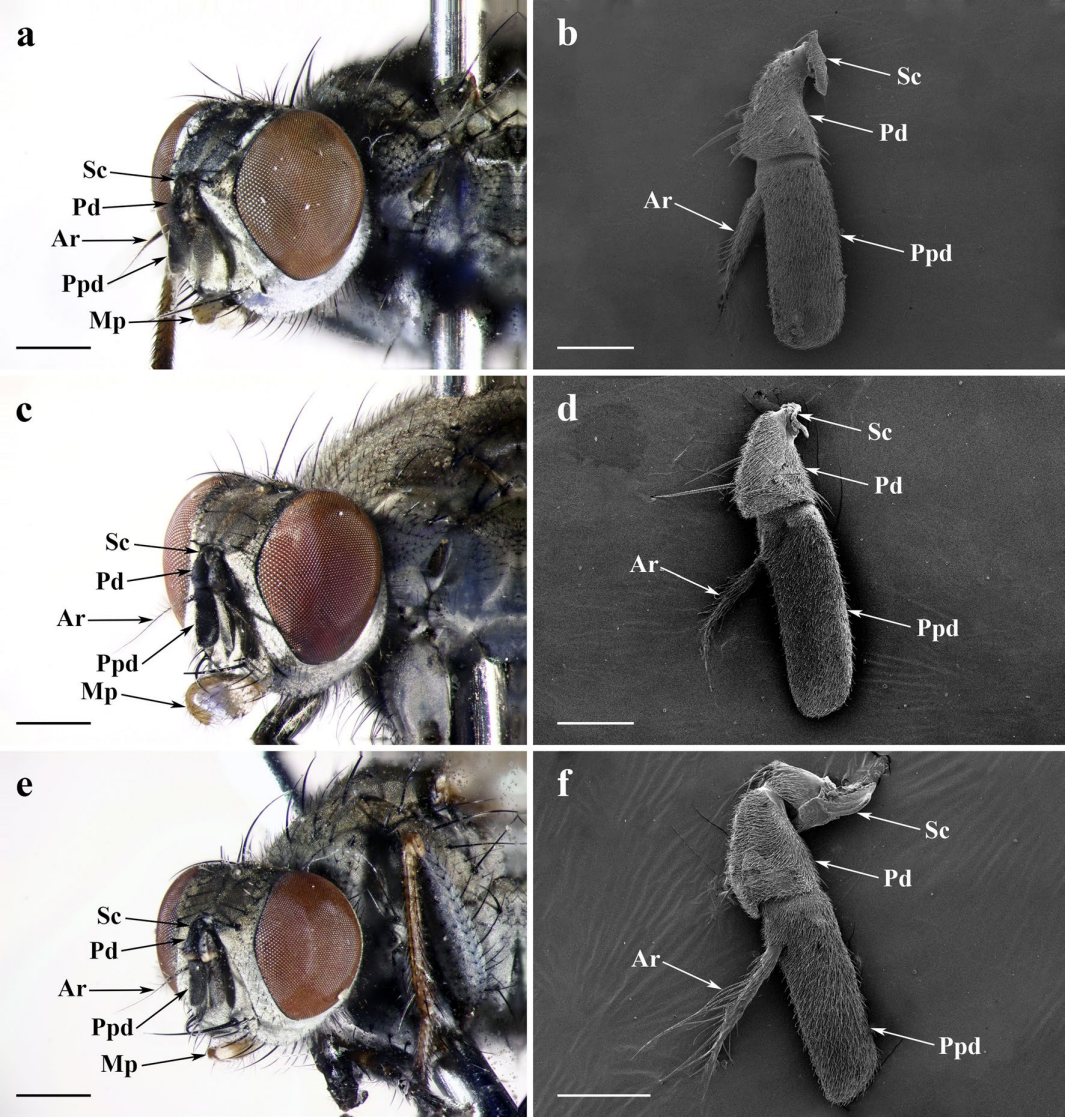
Features on heads and antennae of adult *Lispe longicollis*, *L. orientalis* and *L. pygmaea*. Frontolateral view of (**a**) male *L*. *longicollis*, (**c**) *L*. *orientalis*, and (**e**) *L*. *pygmaea* heads *by stereoscopic microscope*. SEM micrograph of (**b**) male *L*. *longicollis*, (**d**) *L*. *orientalis*, and (**f**) *L*. *pygmaea* antenna, showing the posterior surface. *Ar* arista, *Mp* maxillary palp, *Pd* pedicel, *Ppd* postpedicel, *Sc* scape. Scale bars: (**a,c,e**) = 500 μm; (**b,d,f**) = 150 μm.

*L*. *longicollis*, *L*. *orientalis* and *L*. *pygmaea* all bear a pair of aristate antennae situated at the front of their heads, between two compound eyes. Antennal morphology is composed of three segments: a short proximal scape (Sc), a pedicel (Pd), and a distal flagellum possessing an elongated antennal postpedicel (Ppd) with a slender antennal arista (Ar). A pair of enlarged spoon-like maxillary palps arises at the distal part of the rostrum, a part of the proboscis (Fig. 1a,c,e, Supplementary Fig. S1).

#### Scape and pedicel

**Figure 2:**
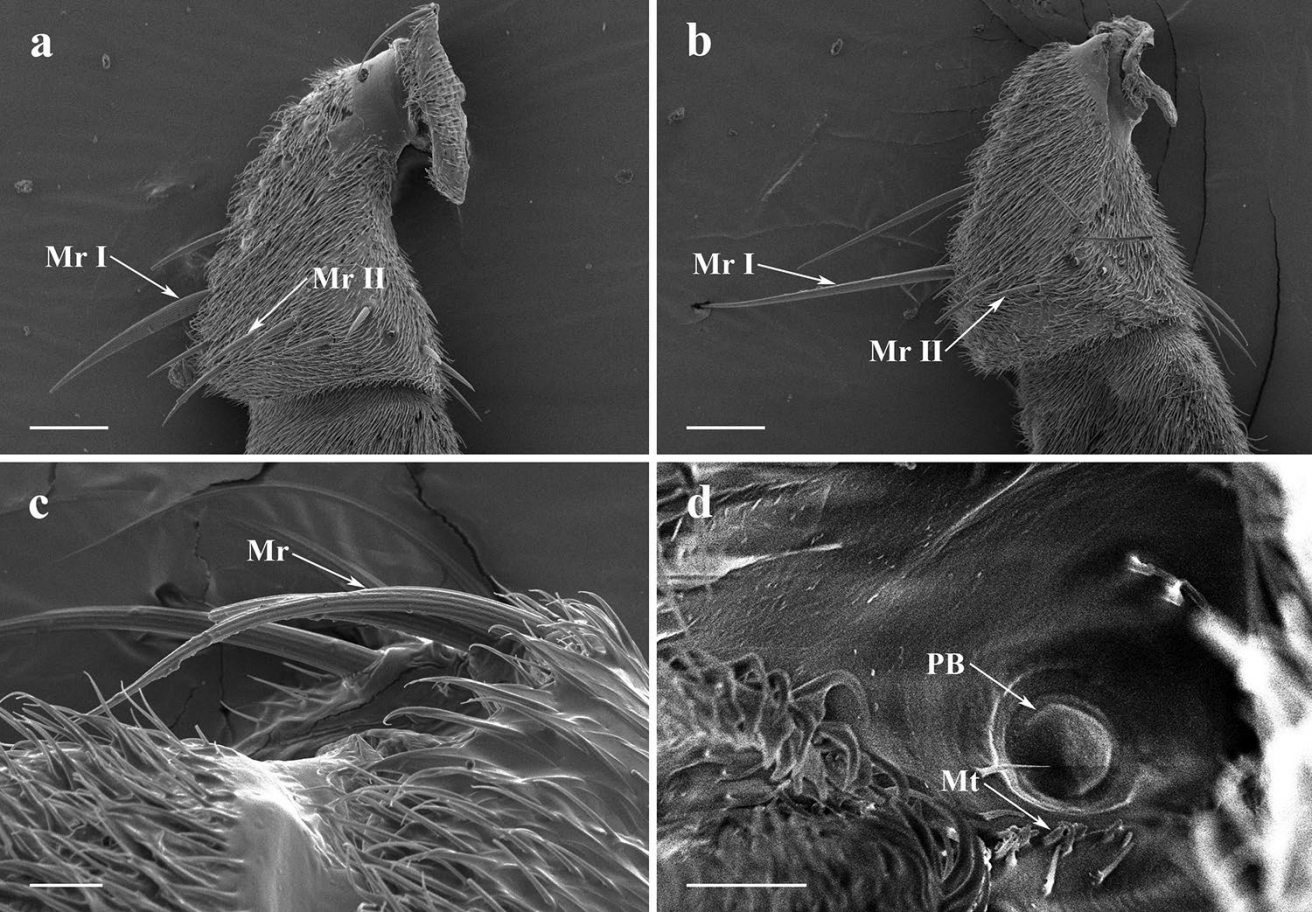
SEM micrographs of features on the antennal scape and pedicel of adult *Lispe longicollis*, *L. orientalis* and *L. pygmaea*. (**a**) Anterior surface of antennal scape and pedicel of male *L*. *longicollis*, arrows showing mechanoreceptors. (**b**) Anterior surface of antennal scape and pedicel of male *L*. *orientalis*, arrows showing mechanoreceptors. (**c**) Mechanoreceptors on antennal scape of male *L*. *pygmaea*. (**d**) Pedicellar button of male *L*. *pygmaea*. *Mr* mechanoreceptors, *Mr I* subtype I mechanoreceptor, *Mr II* subtype II mechanoreceptor, *Mt* microtrichia, *PB* pedicellar button. Scale bars: (**a,b**) 50 μm; (**c**) 10 μm; (**d**) 5 μm.

The antennal scape is the most proximal and the shortest segment (Fig. 1b,d,f), with dense acuminate microtrichia and sporadic cylindrical mechanoreceptors (Mr) with longitudinally grooves (Fig. 2c).

The second segment of the antenna is the antennal pedicel, also covered with microtrichia. Two subtypes of mechanoreceptors can be distinguished by their shape and size on the antennal pedicel (Fig. 2a,b). Usually there are one or two longer mechanoreceptors (Mr I) located on the antennal pedicel. Shorter mechanoreceptors (Mr II) are morphologically like those found on antennal scape, but are straighter in shape and more variable in length.

One pedicellar button (PB) is found in pedicellar recess and near the pedicellar cleft after separated antennal pedicel from antennal postpedicel in *L*. *pygmaea*. Pedicellar button consists of a circular central dome and a slightly convex peripheral ring with a small bunch of peripheral microtrichia (Fig. 2d).

#### Postpedicel

**Figure 3:**
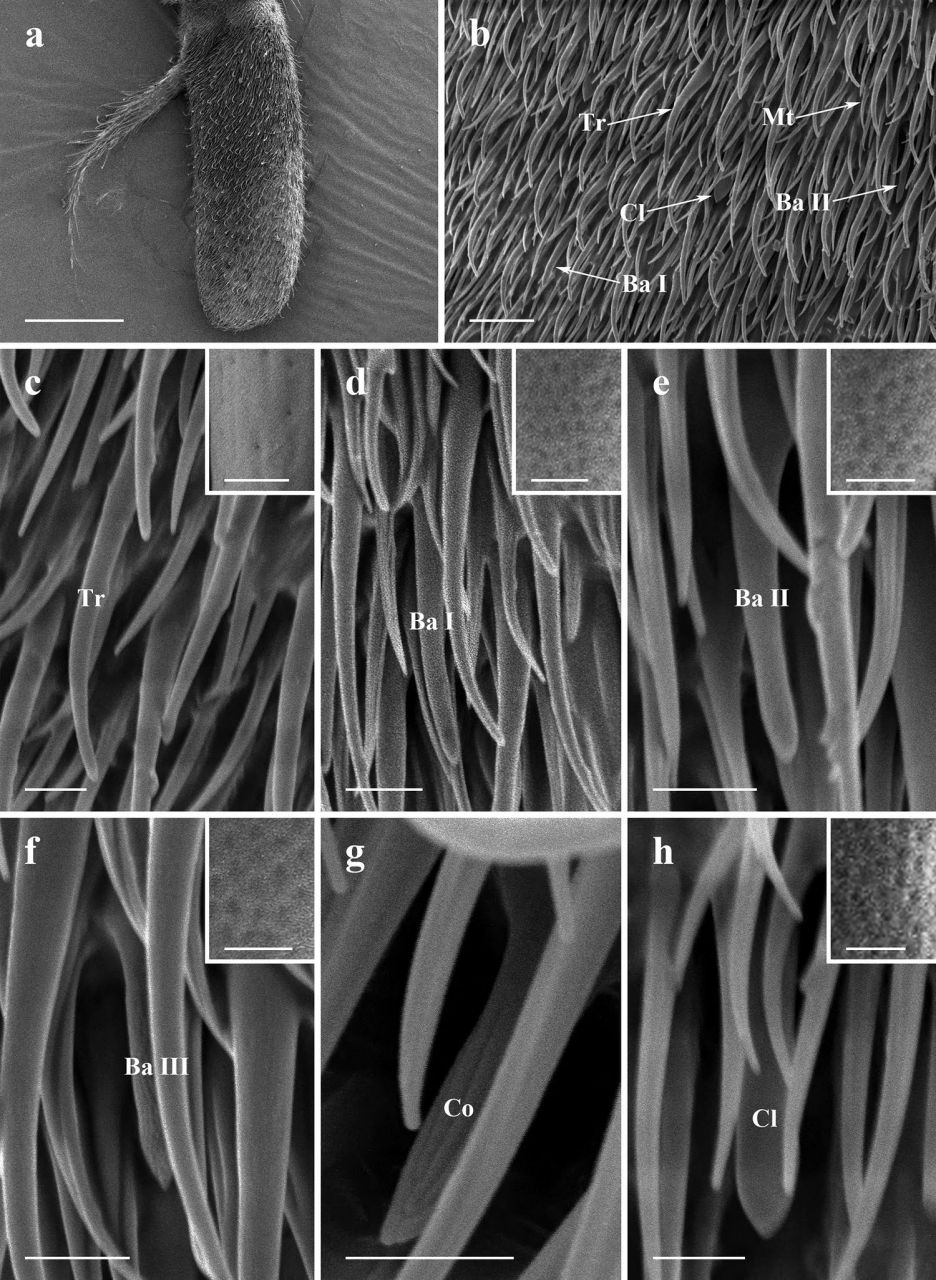
SEM micrographs of features on antennal postpedicel of male *Lispe longicollis*. (**a**) Posterior surface of antennal postpedicel. (**b**) Distribution of different types of sensilla on antennal postpedicel. (**c**) Trichoid sensilla, box showing micropores on the surface. (**d**) Subtype I basiconic sensilla, box showing micropores on the surface. (**e**) Subtype II basiconic sensilla, box showing micropores on the surface. (**f**) Subtype III basiconic sensilla, box showing micropores on the surface. (**g**) Coeloconic sensilla. (**h**) Clavate sensilla, box showing micropores on the surface. *Ba I* subtype I basiconic sensilla, *Ba II* subtype II basiconic sensilla, *Ba III* subtype III basiconic sensilla, *Co* coeloconic sensilla, *Cl* clavate sensilla, *Mt* microtrichia, *Tr* trichoid sensilla. Scale bars: (**a**) 150 μm; (**b**) 10 μm; (**c–f**) 2.5 μm, 0.5 μm in box; (**g**) 2.5 μm.

**Figure 4:**
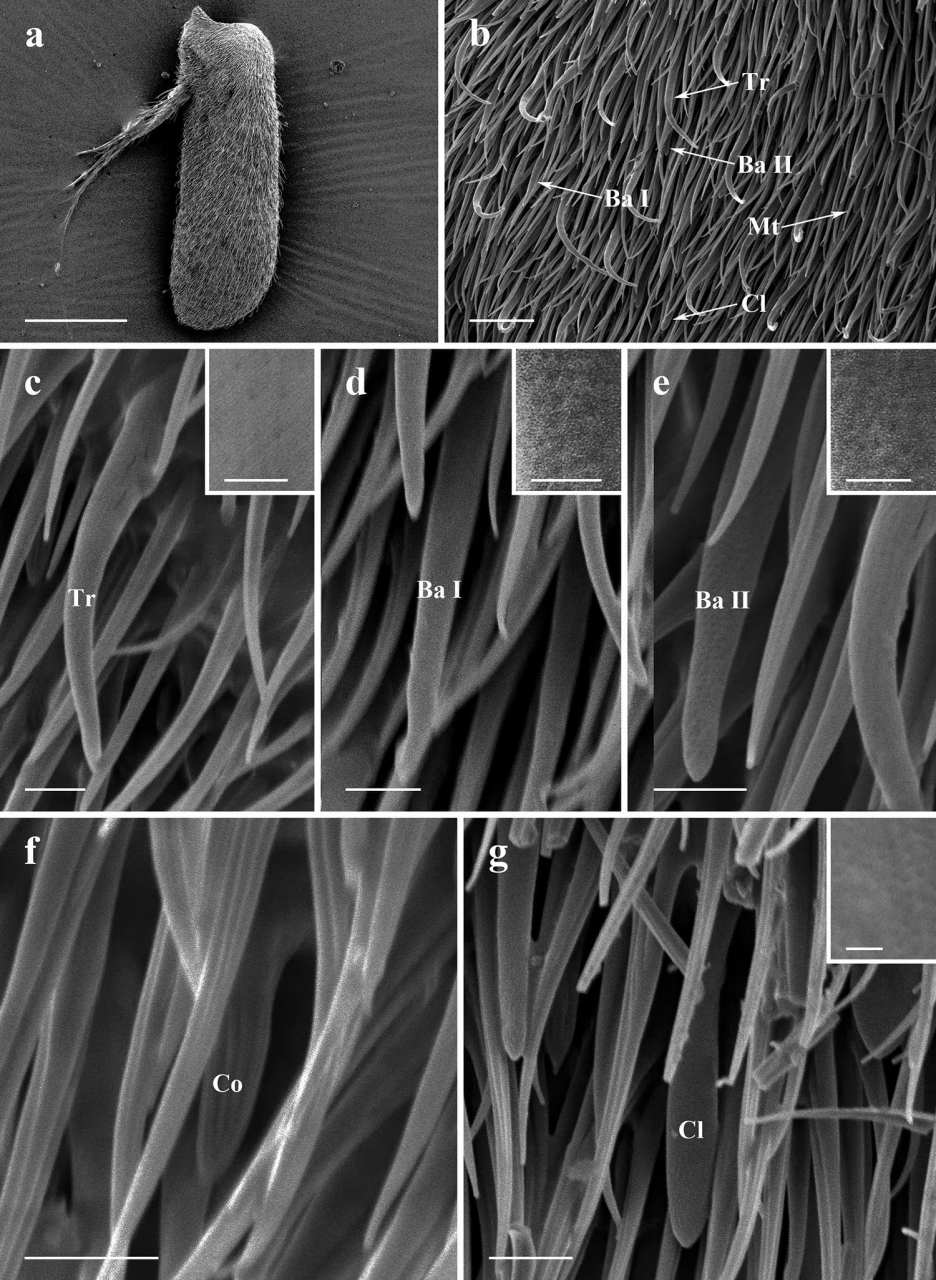
SEM micrographs of features on antennal postpedicel of male *Lispe orientalis*. (**a**) Posterior surface of antennal postpedicel. (**b**) Distribution of different types of sensilla on antennal postpedicel. (**c**) Trichoid sensilla, box showing micropores on the surface. (**d**) Subtype I basiconic sensilla, box showing micropores on the surface. (**e**) Subtype II basiconic sensilla, box showing micropores on the surface. (**f**) Coeloconic sensilla. (**g**) Clavate sensilla, box showing micropores on the surface. *Ba I* subtype I basiconic sensilla, *Ba II* subtype II basiconic sensilla, *Co* coeloconic sensilla, *Cl* clavate sensilla, *Mt* microtrichia, *Tr* trichoid sensilla. Scale bars: (**a**) 150 μm; (**b**) 10 μm; (**c–e,g**) 2.5 μm, 0.5 μm in box; (**f**) 2.5 μm.

**Figure 5:**
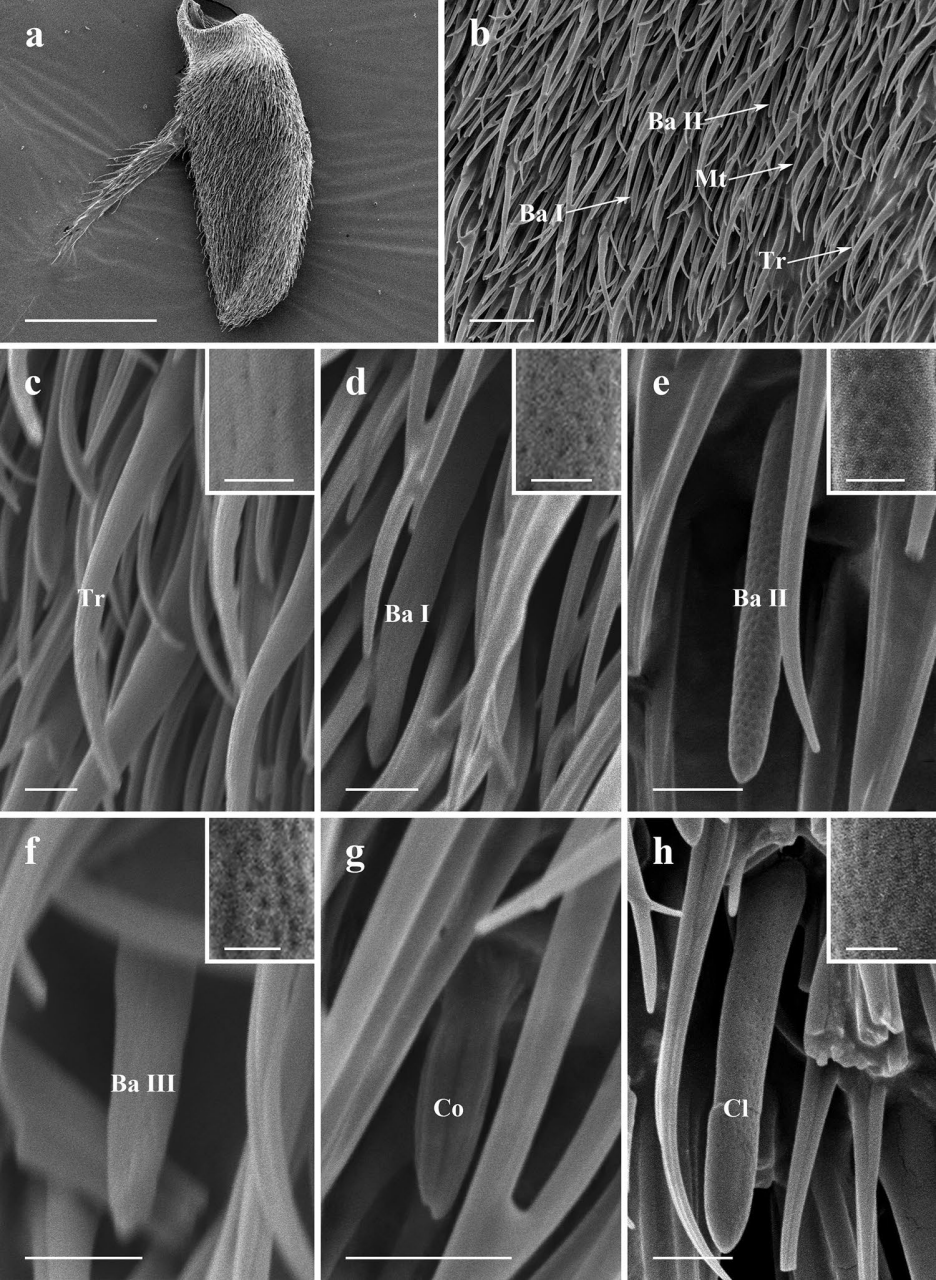
SEM micrographs of features on antennal postpedicel of male *Lispe pygmaea*. (**a**) Posterior surface of antennal postpedicel. (**b**) Distribution of different types of sensilla on antennal postpedicel. (**c**) Trichoid sensilla, box showing micropores on the surface. (**d**) Subtype I basiconic sensilla, box showing micropores on the surface. (**e**) Subtype II basiconic sensilla, box showing micropores on the surface. (**f**) Subtype III basiconic sensilla, box showing micropores on the surface. (**g**) Coeloconic sensilla. (**h**) Clavate sensilla, box showing micropores on the surface. *Ba I* subtype I basiconic sensilla, *Ba II* subtype II basiconic sensilla, *Ba III* subtype III basiconic sensilla, *Co* coeloconic sensilla, *Cl* clavate sensilla, *Mt* microtrichia, *Tr* trichoid sensilla. Scale bars: (**a**) 150 μm; (**b**) 10 μm; (**c–f,h**) 2.5 μm, 0.5 μm in box; (g) = 2.5 μm.

The antennal postpedicel is the most prominent segment of the antenna on which several types of sensilla are found (Figs. 3a,b, 4a,b, 5a,b, Supplementary Fig. S2). It can be divided into two regions, anterior surface, and posterior surface. The surface of antennal postpedicel is covered with dense microtrichia, amongst which four types of sensilla can be found: trichoid sensilla (Tr) (Figs. 3c, 4c, 5c), basiconic sensilla (Ba, subtype I, II and III) (Figs. 3d–f, 4d,e, 5d–f), coeloconic sensilla (Co) (Figs. 3g, 4f, 5g), and clavate sensilla (Cl) (Figs. 3h, 4g, 5h).

#### Maxillary palp

**Figure 6:**
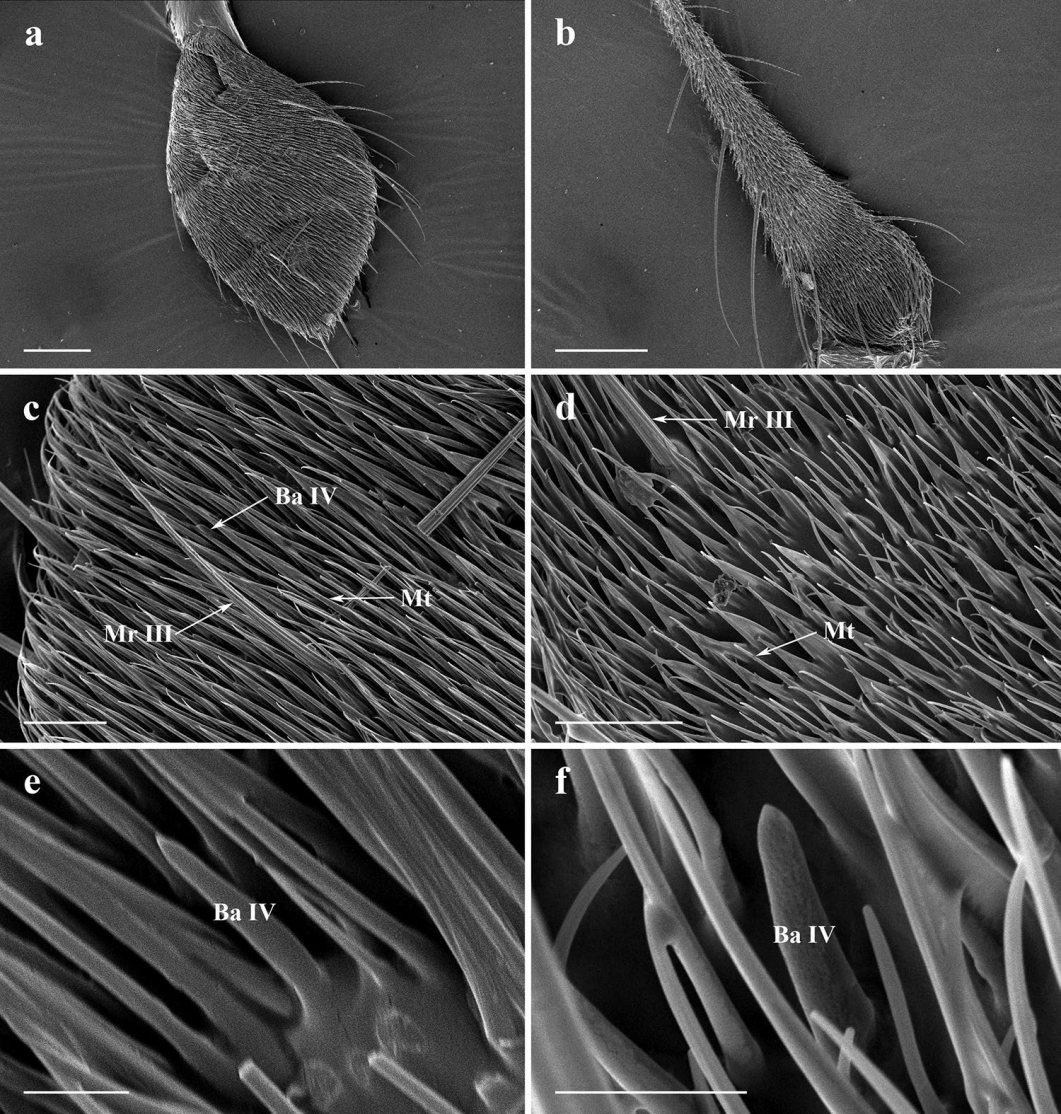
SEM micrographs of features on maxillary palps of *Lispe orientalis* and *L*. *pygmaea*. (**a**) Posterior surface on maxillary palp of male *L*. *orientalis*. (**b**) Posterior surface on maxillary palp of male *L*. *pygmaea*. (**c**) Different types of sensilla on maxillary palp of male *L*. *orientalis*. (**d**) Different types of sensilla on maxillary palp of male *L*. *pygmaea*. (**e**) Subtype IV basiconic sensilla of male *L*. *orientalis*. (**f**) Subtype IV basiconic sensilla of *L*. *pygmaea*. *Mr III* subtype III mechanoreceptor, *Mt* microtrichia, *Ba IV* subtype IV basiconic sensilla. Scale bars: (**a,b**) = 100 μm; (**c,d**) = 20 μm; (**e,f**) = 5 μm.

**Figure 7:**
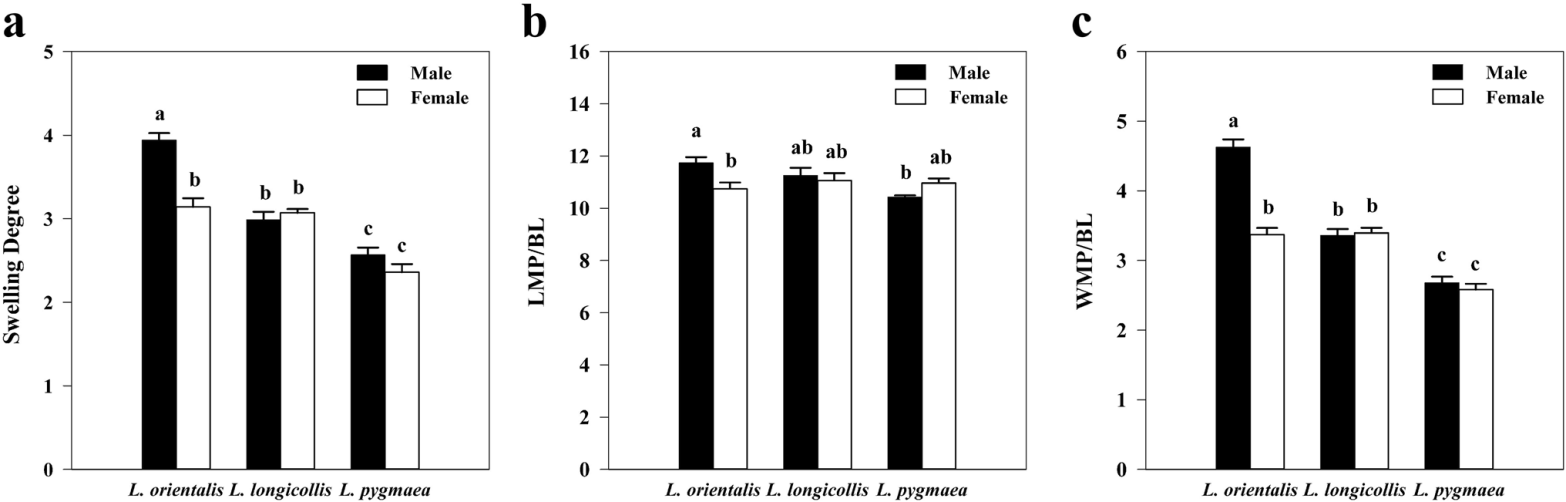
Two-way ANOVA results of characters of maxillary palps among three *Lispe* species and sexes. (**a**) Swelling degree of maxillary palps of the three species among three species and sexes. Male *L. orientalis* has significantly larger swelling than females (*F*_5,24_ = 39.99, *P* < 0.001; species: *F*_2,24_ = 77.05, *P* < 0.001; sex: *F*_1,24_ = 18.96, *P* < 0.001; species × sex: *F*_2,24_ = 13.44, *P* < 0.001). (**b**) The ratio of maxillary palp length to body length (LMP/BL) among three species and two sexes. Male *L. orientalis* has significantly longer maxillary palps than females (*F*_5,24_ = 3.98, *P* = 0.0090; species: *F*_2,24_ = 3.49, *P* = 0.05; sex: *F*_1,24_ = 1.41, *P* = 0.25; species × sex: *F*_2,24_ = 5.75, *P* = 0.0091). (**c**) The ratio of maxillary palp width to body length (WMP/BL) among three species and two sexes. Male *L. orientalis* has significantly wider maxillary palps than females (*F*_5,24_ = 63.58, *P* < 0.001; species: *F*_2,24_ = 111.78, *P* < 0.001; sex: *F*_1,24_ = 34.23, *P* < 0.001; species × sex: *F*_2,24_ = 1.26, *P* < 0.001). Different lower-case letters mean significant differences.

Maxillary palps of males are swollen in the three *Lispe* species, and can be regarded as a representative character of *Lispe*. The ladle-shaped maxillary palps of *L*. *orientalis* with near right-angled edge have the highest degree of swelling among the three species (Figs. 1c, 6a). Comparatively, spoon-shaped maxillary palps of *L*. *longicollis* with a nearly round edge have a lower degree of swelling (Fig. 1a), and that of *L*. *pygmaea* are slightly swollen (Figs. 1e, 6b). The swelling degree of the maxillary palp are significantly different among the three species and between sexes (Table 1, Fig. 7a, *F*_5,24_ = 39.99, *P* < 0.001; species: *F*_2,24_ = 77.05, *P* < 0.001; sex: *F*_1,24_ = 18.96, *P* < 0.001; species × sex: *F*_2,24_ = 13.44, *P* < 0.001), and much larger than typical Muscidae species such as *Musca domestica* and *Fannia hirticeps* (Table 1).

**Table 1:** Length, width, swelling degree (10^–1^ × width/length) of maxillary palps, body length and the ratio of length and width of maxillary palps to body length (10^–2^ × LMP/BL and 10^–2^ × WMP/BL) in three *Lispe* species, *Musca domestica*, and *Fannia hirticeps* (μm ± SD, n = 5).

Species	Sex	Length	Width	Swelling degree	Body length	LMP/BL	WMP/BL
*Lispe orientalis*	M	746.35 ± 30.38	293.82 ± 5.31	3.94a	6360.88 ± 269.08	11.73a	4.62a
	F	760.73 ± 55.25	239.24 ± 26.58	3.14b	7082.89 ± 486.31	10.74b	3.38b
*L*. *longicollis*	M	741.38 ± 31.17	221.14 ± 12.34	2.98b	6590.10 ± 201.83	11.25ab	3.36b
	F	751.09 ± 45.83	230.55 ± 15.17	3.07b	6806.50 ± 597.80	11.03ab	3.39b
*L*. *pygmaea*	M	554.95 ± 28.58	142.29 ± 8.80	2.56c	5321.77 ± 288.54	10.43b	2.67c
	F	628.52 ± 39.45	148.25 ± 14.68	2.36c	5737.99 ± 373.96	10.95ab	2.38c
*Musca domestica* (Smallegange et al. 2008)^2^	−	495	72	1.45d	−	−	−
*Fannia hirticeps* (Wang et al. 2012)^25^	−	360	40	1.11d	−	−	−

*M* male, *F* female, *BL* body length, *LMP* length of maxillary palp, *WMP* width of maxillary palp, − undetermined.

Different lower-case letters on swelling degree data mean statistically significantly different (P < 0.05, n = 5).

Our results show that compared to their body length, the relative maxillary palp length and the relative width are different between sex and among these *Lispe* species (Fig. 7b,c). There are significant differences in the ratio of maxillary palp length to body length (LMP/BL) (Fig. 7b, species: *F*_2,24_ = 3.49, *P* = 0.05; sex: *F*_1,24_ = 1.41, *P* = 0.25; species × sex: *F*_2,24_ = 5.75, *P* = 0.0091) and the ratio of maxillary palp width to body length (WMP/BL) among three species and between sex (Fig. 7c, species: *F*_2,24_ = 111.78, *P* < 0.001; sex: *F*_1,24_ = 34.23, *P* < 0.001; species × sex: *F*_2,24_ = 1.26, *P* < 0.001). These results showed strong sexual dimorphism of swelling degree (post hoc test, *L*. *orientalis*: *P* < 0.001, *L*. *longicollis*: *P* = 0.507, *L*. *pygmaea*: *P* = 0.103), LMP/BL (post hoc test, *L*. *orientalis*: *P* = 0.005, *L*. *longicollis*: *P* = 0.548, *L*. *pygmaea*: *P* = 0.108), and WMP/BL (post hoc test, *L*. *orientalis*: *P* < 0.001, *L*. *longicollis*: *P* = 1.000, *L*. *pygmaea*: *P* = 0.975) in *L*. *orientalis* but not in other species.

Two types of sensilla are found on the maxillary palps: mechanoreceptors and subtype IV basiconic sensilla (Ba IV). Mechanoreceptors (Mr III) are distributed around the distal rim of the maxillary palp (Fig. 6a–d), and Ba IV are blunt-tipped (Fig. 6e,f), distributed amongst dense microtrichia.

### Sensilla on antennal postpedicel

#### Trichoid sensilla

Trichoid sensilla (Tr) are the most conspicuous and the most numerous sensilla in all three *Lispe* species. They gradually taper from relatively thick base to an acute apex, with micropores on the cuticle surface (Figs. 3c, 4c, 5c). Tr are the longest and with the largest basal diameter among all four types of sensilla on antennal postpedicel, about 20–25 μm in length (Table 2). Densities of Tr increase from the proximal region towards distal region on both anterior surface and posterior surface of antennal postpedicel (Table 3).

**Table 2:** Length, basal diameter, and tip diameter (Cl only) of sensilla on antennal postpedicel of three *Lispe* species (μm ± SD, n = 10).

Species	Type	Length	Basal diameter	Tip diameter
*Lispe orientalis*	Tr	22.09 ± 0.87	1.94 ± 0.09	−
	Ba I	14.60 ± 0.89	1.56 ± 0.31	−
	Ba II	11.28 ± 0.65	1.38 ± 0.20	−
	Co	4.45 ± 0.23	1.32 ± 0.11	−
	Cl	12.02 ± 0.03	1.51 ± 0.07	2.32 ± 0.09
*L*. *longicollis*	Tr	19.36 ± 0.63	1.78 ± 0.06	−
	Ba I	12.75 ± 0.56	1.50 ± 0.11	−
	Ba II	10.27 ± 0.45	1.39 ± 0.12	−
	Ba III	7.09 ± 0.74	1.35 ± 0.10	−
	Co	4.41 ± 0.98	1.28 ± 0.44	−
	Cl	12.33 ± 0.81	1.51 ± 0.17	2.25 ± 0.26
*L*. *pygmaea*	Tr	25.68 ± 0.84	1.95 ± 0.09	−
	Ba I	14.71 ± 0.69	1.33 ± 0.07	−
	Ba II	10.01 ± 0.57	1.46 ± 0.12	−
	Ba III	5.96 ± 0.69	1.06 ± 0.18	−
	Co	3.57 ± 0.25	0.92 ± 0.04	−
	Cl	12.53 ± 0.01	1.53 ± 0.03	1.98 ± 0.22

*Ba I* basiconic sensilla I, *Ba II* basiconic sensilla II, *Ba III* basiconic sensilla III, *Cl* clavate sensilla, *Co* coeloconic sensilla, *Tr* trichoid sensilla, − undetermined.

**Table 3:** Average density of sensilla (10^−3^ μm^−2^ ± SD, n = 10) on antennal postpedicel of three *Lispe* species.

Species	Type	Anterior surface				Posterior surface			
		Proximal	Median	Distal	Average	Proximal	Median	Distal	Average
*Lispe orientalis*	Tr	4.10 ± 1.17	5.61 ± 2.14	7.52 ± 2.38	5.74 ± 1.89	2.31 ± 1.79	6.08 ± 1.88	5.90 ± 1.43	4.76 ± 1.71
	Ba	3.47 ± 2.46	2.36 ± 1.92	0.00	1.94 ± 1.46	2.31 ± 1.42	2.95 ± 3.17	0.45 ± 0.94	1.90 ± 1.82
	Co	0.00	0.26 ± 0.85	0.29 ± 1.00	0.19 ± 0.61	0.00	0.69 ± 1.21	0.00	0.23 ± 0.40
	Cl	1.58 ± 2.12	0.26 ± 0.64	0.00	0.61 ± 0.92	0.87 ± 1.45	0.52 ± 1.17	0.00	0.46 ± 0.84
*L*. *longicollis*	Tr	7.73 ± 3.95	14.34 ± 3.12	17.88 ± 1.84	13.32 ± 2.96	5.90 ± 3.29	10.57 ± 2.12	16.55 ± 4.01	11.01 ± 3.14
	Ba	2.10 ± 2.21	4.45 ± 1.92	3.30 ± 2.23	3.28 ± 2.12	1.22 ± 1.17	3.16 ± 2.03	6.35 ± 1.79	3.58 ± 1.64
	Co	0.00	0.25 ± 0.69	0.00	0.08 ± 0.23	0.00	0.16 ± 0.52	0.00	0.05 ± 0.17
	Cl	2.17 ± 2.19	0.64 ± 1.00	0.35 ± 0.73	1.05 ± 1.31	0.52 ± 0.84	0.32 ± 0.70	0.00	0.28 ± 0.52
*L*. *pygmaea*	Tr	5.01 ± 2.03	10.10 ± 2.55	13.31 ± 3.78	9.47 ± 2.79	2.78 ± 1.98	7.64 ± 1.46	11.28 ± 2.49	7.23 ± 1.95
	Ba	2.93 ± 2.04	3.95 ± 2.91	1.35 ± 1.69	2.74 ± 2.20	3.13 ± 3.34	3.13 ± 1.79	2.26 ± 1.43	2.84 ± 2.16
	Co	0.00	0.16 ± 0.52	0.19 ± 0.58	0.12 ± 0.37	0.00	0.45 ± 0.96	0.45 ± 1.43	0.30 ± 0.80
	Cl	0.87 ± 1.47	0.00	0.00	0.29 ± 0.48	2.08 ± 0.78	0.35 ± 0.73	0.00	0.81 ± 0.54

*Ba* basiconic sensilla, *Cl* clavate sensilla, *Co* coeloconic sensilla, *Tr* trichoid sensilla.

#### Basiconic sensilla

Three subtypes of basiconic sensilla (Ba) are identified on antennal postpedicel according to their shape and size. Subtype I basiconic sensilla (Ba I) are shorter than Tr, about 12–14 μm in length (Table 2). They appear as sturdy pegs that gradually taper to an acute tip (Figs. 3d, 4d, 5d). Subtype II basiconic sensilla (Ba II) are pegs with blunt-tip (Figs. 3e, 4e, 5e), about 10–12 μm in length, shorter than Ba I (Table 2). In *L*. *longicollis* and *L*. *pygmaea*, subtype III basiconic sensilla (Ba III) are also identified on the surface of antennal postpedicel (Figs. 3f, 5f). Compared with Ba I and Ba II, Ba III are the smallest both in length and basal diameter (Table 2). Ba are distributed relatively evenly on the surface of antennal postpedicel, less dense than Tr (Table 3).

#### Coeloconic sensilla

Coeloconic sensilla (Co) are characterised by longitudinally grooved walls, projecting from a shallow depression of integument. They are typically cone-shaped with sharp tips (Figs. 3g, 4f, 5g). Coeloconic sensilla are about 3–4 μm in length, much smaller compared to other types of sensilla (Table 2), and scattered sparsely on the surface of antennal postpedicel (Table 3).

The size and density of Co among different muscoid species of six genera (*Hydrotaea armipes* Fallén, *Musca domestica* L., *Scathophaga stercoraria* L., *Delia radicum* L., *D*. *floralis* Fallén, *D*. *antiqua* Meigen, *D*. *platura* Meigen, *Fannia hirticeps* Stein, *F*. *scalaris* Fabricius, *F*. *canicularis* L.) are compared in Tables 3 and 4. The sizes of Co on antennal postpedicel of these *Lispe* species are like other muscoid species, but the average densities of Co on their antennal postpedicel are lower.

**Table 4:** Length, basal diameter (μm ± SD) and average density (10^−3^ μm^−2^ ± SD) of coeloconic sensilla on antennal postpedicel of muscoid species.

Family	Species	Sex	Length	Basal diameter	Anterior surface density	Posterior surface density
Muscidae	*Lispe orientalis*	M	4.45 ± 0.23	1.32 ± 0.11	0.19 ± 0.61	0.23 ± 0.40
	*L*. *longicollis*	M	4.41 ± 0.98	1.28 ± 0.44	0.08 ± 0.23	0.05 ± 0.17
	*L*. *pygmaea*	M	3.57 ± 0.25	0.92 ± 0.04	0.12 ± 0.37	0.30 ± 0.80
	*Hydrotaea armipes* (Wang et al. 2014)^31^	M	3.20 ± 0.92	1.32 ± 0.15	×	×
	*Musca domestica* (Smallegange et al. 2008)^2^	−	1.5–3.0	0.6–0.9	×	×
Scathophagidae	*Scathophaga stercoraria* (Liu et al. 2016)^32^	M	3.65 ± 1.17	1.35 ± 0.52	0.57 ± 0.39	1.33 ± 0.58
		F	2.67 ± 0.18	0.93 ± 0.06	0.76 ± 0.57	0.50 ± 0.19
Anthomyiidae	*Delia radicum* (Ross 1992)^18^	M	4.0 ± 0.6	1.3 ± 0.3	0.39 ± 0.07	0.30 ± 0.02
		F			0.28 ± 0.13	0.27 ± 0.08
	*D*. *floralis* (Ross 1992)^18^	M	4.8 ± 0.3	1.8 ± 0.3	0.34 ± 0.18	0.20 ± 0.01
		F			0.23 ± 0.15	0.30 ± 0.05
	*D*. *antiqua* (Ross 1992)^18^	M	4.5 ± 0.6	1.6 ± 0.2	0.32 ± 0.07	0.41 ± 0.04
		F			0.35 ± 0.19	0.40 ± 0.07
	*D*. *platura* (Ross 1992)^18^	M	2.9 ± 0.2	1.2 ± 0.3	0.50 ± 0.05	0.23 ± 0.09
		F			0.30 ± 0.09	0.30 ± 0.24
Fanniidae	*Fannia hirticeps* (Wang et al. 2012)^25^	M	4.67 ± 0.82	1.49 ± 0.18	1.2 ± 0.3	1.6 ± 0.4
	*F*. *scalaris* (Zhang et al. 2013)^10^	M	2.40 ± 0.42	1.11 ± 0.11	1.7 ± 1.0	3.0 ± 0.8
	*F*. *canicularis* (Zhang et al. 2013)^10^	M	3.15 ± 0.14	0.49 ± 0.18	1.6 ± 0.9	1.6 ± 0.9

*M* male, *F* female, − unidentified; × no data.

#### Clavate sensilla

Clavate sensilla (Cl) can be distinguished by distal club-like swelling (Figs. 3h, 4g, 5h), about 12 μm in length, shorter than trichoid sensilla (Table 2). The distribution of Cl is relatively aggregated, most of them are discovered on the proximal and middle region of antennal postpedicel surface (Table 3).

## Discussion

The present study describes the antennal sensilla of three aquatic predators, *L*. *longicollis*, *L*. *orientalis* and *L*. *pygmaea* using scanning electron microscopy. The morphology and distribution of mechanoreceptors, pedicellar button, trichoid sensilla, basiconic sensilla, and clavate sensilla of three *Lispe* species resemble to previous results on *L*. *neimongola*^9^ and other muscoid species, such as *Delia radicum* L.^18^, *Musca domestica* L.^2^, *Fannia hirticeps* Stein^25^, and *Scathophaga stercoraria* L.^26^. Mechanoreceptors are known to be sensitive to physical stimuli like gravity, air vibration, and tension caused by muscle activity^27^. Micropores were detected on the surface of Tr, Ba, and Cl (Figs. 3, 4, 5 boxes), which are characteristic in chemoreceptors. In addition, electrophysiological^28,29^ and neurological^30,31^ studies also identified odorant receptors (OR) and gustatory receptors (GR) in Tr^30,32,33^, Ba^30,34,35^, Co^33,36^, confirming their olfactory function.

The swollen maxillary palps in *Lispe* may increase their chemosensory functions. Different from the club-like maxillary palps in most of other fly species, maxillary palps of *Lispe* species are significantly swollen and flattened, and swelling degrees of maxillary palps in the three *Lispe* species are generally larger than typical muscoid species, such as *Musca domestica* L.^2^ and *Fannia hirticeps* Stein^25^ (Table 1). Maxillary palps mainly acted as gustatory sensory organ^37^ that react to molecules with low or zero vapor pressure, involved in contact or short-distance chemosensory functions^38^, compared to that of antennae, which typically perceive more volatile olfactory signals or chemical cues^39^. Shiraiwa^40^ pointed out that maxillary palps of fruit flies can improve their sensitivity to food odours, others suggested that maxillary palps perceive olfactory signals at shorter distance, and can be integrated with the signals perceived in antennae to allow better manoeuvring when approaching lures^41^. Larger maxillary palps of *L*. *neimongola* were suggested to provide larger surface area for basiconic sensilla and enhance the perception of gustatory odours or signals^9^. For predators flying rapidly to chase prey like *Lispe*^22,42^, more sensilla can increase their behavioural responsiveness^43^. The swollen maxillary palps of the three *Lispe* species may function similarly to improve their gustatory and/or olfactory sensitivity.

Maxillary palps could also be a signaller as well as a signal receiver. In respond to higher selection pressure of searching for food, hosts, or oviposition sites by chemosensory, female insects usually have larger antennae and maxillary palps or more sensilla attached to them^9,44,45^. In *L*. *orientalis* and *L*. *neimongola*^9^, maxillary palps are significantly more swollen in males than in females. Light microscopy photos (Figs. 1c, Supplementary Fig. S1c) and field observations show that maxillary palps of male *L*. *orientalis* are more conspicuous than other species when observed from a distance. Empiracle evidence shows that during courtship dance, some male *Lispe* flies circle around the female and flash their maxillary palps^21,42^. This could be important in correct species recognition and successful copulation, as many *Lispe* flies have highly overlapped habitats and ecological niches^42,46^. Thus, the maxillary palps could be dual-functional for male *Lispe* flies, and this is among some rare cases that an olfactory organ also plays a role as chemical signal receiver and as visual signal conveyer, which also indicates maxillary pales of *L*. *orientalis* are under different levels of sexual selection pressure.

Coeloconic sensilla are common on antennal postpedicel in most other fly species^8,19,25,26,47,48^, but are relatively sparse on antennal postpedicel of these three *Lispe* species, even completely missing in *L*. *neimongola*^9^. Beside chemosensory function, Coeloconic sensilla have been also proved to be sensitive to temperature and/or humidity signals^49,50^. Compared with olfactory, temperature or humidity, predators rely more on acute vision which enhance their ability of colour vision, movement awareness and depth perception, especially on fast moving preys^19,51^. Lower number of coeloconic sensilla on antennal postpedicel reflect the adaptation to predatory lifestyle and could be regarded as a character of the genus *Lispe*.

## Methods

Adult *L*. *longicollis*, *L*. *orientalis* and *L*. *pygmaea* were captured from Kalamaili Ungulate Nature Reserve, Xinjiang, China, in August 2013. All specimens were pinned as museum samples and air dried on site. Morphology of antennae and maxillary palps were examined under Olympus SZX16 stereoscopic microscope (Olympus Corp., Tokyo, Japan), morphological photographs were taken by a Canon 500D digital camera (Canon, Inc., Tokyo, Japan) coupled with stereoscopic microscope. Continuous images on different focal lengths were composed by Helicon Focus for Windows (Helicon Soft Ltd., Kharkov, Ukraine). Five specimens for both sexes of each species were used for measuring body length as well as length and width of maxillary palp. Three male specimens for each species were used for scanning electron microscopy. Heads of all specimens were cut off, then surface debris was removed by rinsing in phosphate buffered saline buffer (pH 7.4). Subsequently, antennae and maxillary palps were dissected respectively, cleaned with detergent by ultrasonic cleaner. After dehydration in graded ethanol series (twice 15 min each with 60%, 70%, 80%, 90%, 95%, 100% ethanol), antennae and maxillary palps were mounted on aluminium stubs with double-sided adhesive tape, then left in a desiccator for 24 h to dry thoroughly. Samples were coated with gold and observed with a HITACHI S34Q scanning electron microscopy (Hitachi Corp., Tokyo, Japan) at the Microscopy Core Facility, Biological Technology Centre, Beijing Forestry University (Beijing, China).

Length, width of maxillary palps and body length of five specimens for each sex were measured. Then the swelling degree (width to length) of maxillary palps and the ratio of maxillary palp measures to body length (length of maxillary palp to body length and width of maxillary palp to body length) were calculated and compared by two-way ANOVA in SPSS 22.0 (IBM Corp., Armonk, New York) between different species and different sexes of each species. Results of two-way ANOVA were visualized by Sigmaplot 12.5 (Systat Software, Inc., Chicago, Illinois). Length, basal diameter, tip diameter (clavate sensilla only), density and distribution of sensilla were measured using micrographs taken under different magnifications. The length of each single sensillum was measured (ten repeats of each type of sensilla) from the proximal rim to the tip. Density and distribution of various types of sensilla were measured by measuring square areas (each representing 576 µm^2^) from proximal, median, and distal part (each consists one third of the antenna in length) of the antenna on both sides^2^, and ten quadrates were measured for each part. In this study, the terminology applied to describe antennal morphology and classification of types of sensilla followed those used by Cumming and Wood^52^.

## Supplementary Information

Supplementary Information.

Supplementary Figure S1.

Supplementary Figure S2.

## Data Availability

All data generated or analysed during this study are included in this published article.
