# Author Correction: Antennal and palpal sensilla of three predatory *Lispe* species (Diptera: Muscidae): an ultrastructural investigation

**DOI:** 10.1038/s41598-022-04882-z

**Published:** 2022-01-06

**Authors:** Genting Liu, Qike Wang, Xianhui Liu, Xinyu Li, Xiunan Pang, Dong Zhang

**Affiliations:** 1 School of Ecology and Nature Conservation, Beijing Forestry University, Qinghua East Road No. 35, Mailbox 162, Beijing, 100083 China; 2 School of BioSciences, The University of Melbourne, Victoria, 3010 Australia; 3 University of California Davis, Davis, CA 95616 USA

Correction to: *Scientific Reports* 10.1038/s41598-021-97677-7, published online 15 September 2021

In the original version of this Article, Affiliations 1, 2 and 3 were not listed in the correct order. The correct affiliations are listed below:

Affiliation 1:

School of Ecology and Nature Conservation, Beijing Forestry University, Qinghua East Road No. 35, Mailbox 162, Beijing, 100083, China.

Affiliation 2:

School of BioSciences, The University of Melbourne, Victoria, 3010, Australia.

Affiliation 3:

University of California Davis, Davis, CA, 95616, USA.

The original Article has been corrected.

