# Supplementary material for: Antennal and palpal sensilla of three predatory Lispe species (Diptera: Muscidae): an ultrastructural investigation
Source: Sci Rep. 2021 Sep 15;11:18357. doi: 10.1038/s41598-021-97677-7 (PMC8443604; doi:10.1038/s41598-021-97677-7)
Supplement: Supplementary file 1 — Supplementary Information. [file 41598_2021_97677_MOESM1_ESM.docx]

**Supplementary Figure captions**

**Figure S1. Features on heads and antennae of adult *Lispe* *longicollis*, *L.* *orientalis* and *L. pygmaea*.** Frontolateral view of (a) female *L*. *longicollis*, (c) *L*. *orientalis*, and (e) *L*. *pygmaea* heads **by stereoscopic microscope**. SEM micrograph of (b) female *L*. *longicollis*, (d) *L*. *orientalis*, and (f) *L*. *pygmaea* antenna, showing the anterior surface. Abbreviations: Ar, arista; Mp, maxillary palp; Pd, pedicel; Ppd, postpedicel; Sc, scape. Scale bars: a, c, and e = 500 μm; b, d, and f = 150 μm.

**Figure S2.** **SEM micrographs of features on antennal postpedicel of female *Lispe* *longicollis*, *L.* *orientalis* and *L. pygmaea*.** Different types of sensilla on antennal postpedicel of (a) *L*. *longicollis*, (c) *L*. *orientalis*, and (f) *L*. *pygmaea*. Magnification of (b) trichoid sensilla, (d) subtype I basiconic sensilla, (e) subtype II basiconic sensilla, (g) subtype III basiconic sensilla, and (h) coeloconic sensilla. Abbreviations: Ba I, subtype I basiconic sensilla; Ba II, subtype II basiconic sensilla; Ba III, subtype III basiconic sensilla; Co, coeloconic sensilla; Cl, clavate sensilla; Mt, microtrichia; Tr, trichoid sensilla. Scale bars: a, c, and f = 10 μm; b and d = 2 μm; e, g, and h = 1 μm.
